# Editorial: *In vitro* and *in vivo* models for neurodevelopmental disorders

**DOI:** 10.3389/fnins.2023.1239577

**Published:** 2023-07-11

**Authors:** Angelica D'Amore, Maria Marchese, Wardiya Afshar-Saber, Mustafa Q. Hameed

**Affiliations:** ^1^Department of Neurology, Kirby Neurobiology Center, Boston Children's Hospital and Harvard Medical School, Boston, MA, United States; ^2^Department of Molecular Medicine, IRCCS Stella Maris Foundation, Pisa, Italy

**Keywords:** neurodevelopmental disorders (NDDs), Alzheimer's disease, animal model, induced pluripotent stem cells (IPSCs), ARSs

Neurodevelopmental disorders (NDDs) are a group of disorders affecting brain development and function. Each disorder within this heterogenous group (i.e., intellectual disability/ID, autism spectrum disorder/ASD, attention-deficit/hyperactivity disorder etc.) has distinct clinical characteristics and phenotypical variability. While each disorder is indeed defined by a set of symptoms, individual symptoms are not necessarily restricted to one disorder. Comorbidity of two or more NDDs is frequently observed: for instance, a combination of ID, ASD, and epilepsy is commonly reported in patients (Parenti et al., 2020). Many NDDs have strong genetic bases and several hundred genes have been implicated in such NDDs either through genetic association studies, rare mutations, copy number variation etc. However, there is a significant proportion of NDDs with an unknown genetic cause (i.e., idiopathic) and, in those instances, the diagnosis is based only on interviews and medical examination. To date, several pathways have been associated with NDDs (mTOR, WNT, pathways associated with chromatin remodeling and synaptic function etc.) and understanding the molecular mechanism behind NDDs has the potential to define druggable targets, making *in-vitro* and *in-vivo* disease models fundamental tools for advancing the field (Thapar et al., 2017; Cardoso et al., 2019; Bozzi and Fagiolini, 2020; Nussinov et al., 2023).

In the last decades, the scientific community has been focusing on investigating the cellular and molecular mechanisms behind NDDs, trying to develop effective tools, using both *in-vitro* and *in-vivo* models. These complex disorders can be modeled using either animal models, such as rodents and zebrafish, or cellular models like iPSCs, enabling behavioral and functional analyses in the presence of disease-causing mutations. While existing models have allowed for a deeper and better understanding of the molecular and cellular mechanisms that are disrupted in NDDs, most of these studies focus on isolated variants and do not account for fundamental evolutionary differences between animals and humans, especially during development (Bozzi and Fagiolini, 2020; Sabitha et al., 2020; Díaz-Caneja et al., 2021). Using human cell-based and animal models in tandem is crucial for a better understanding of the mechanisms leading to the onset and progression of NDDs. Identifying the specifics and correlations between NDDs and phenotypes observed in these models will help the scientific community in developing new therapeutic strategies.

In this Research Topic, we present a collection of research and review articles that address the importance of *in-vitro* and *in-vivo* modeling in expanding the understanding on neurodevelopment and NDDs. The collection contains two original research articles and two review articles.

The first original research article by Hingorani et al. investigates the importance of the serotonergic system in the brain development using an *in-vitro* model of rat glial cells and mouse midbrain neurons co-cultures and monolayers. Individual serotonergic axons (which produce highly stochastic trajectories, fundamental to the construction of regional fiber densities) were studied with a set of complementary high-resolution methods: confocal microscopy, holotomography (refractive index-based live imaging), and super-resolution (STED) microscopy, making this the first high-resolution analysis of single serotonergic axons *in vitro*.

The following original research article by Haukedal et al. explores disease phenotypes specific to glutamatergic forebrain neurons in Alzheimer's Disease (AD), implementing familial and sporadic human induced iPSCs models as well as the 5xFAD mouse model (). Despite AD is not an NDD, the use of iPSCs in this model is a good example on how iPSCs can help investigating the longitudinal progression of the clinical characteristics observed in patients and modeled in cells. In this paper, after recapitulating characteristic late AD phenotypes, such as increased Aβ secretion and Tau hyperphosphorylation, the authors were able to identify Golgi fragmentation as one of the earliest AD phenotypes, indicating potential impairments in protein processing and post-translational modifications. Furthermore, Golgi fragmentation was exacerbated via additional risk variants in *SORL1*, a regulator of endosomal traffic and recycling in human neurons. These findings were confirmed both *in-vitro* and *in-vivo*, clearly indicating that Golgi fragmentation is a universal perturbation in both familial early- and sporadic late-onset AD, validating the clinical relevance of these findings.

The two review articles focus respectively, on dominant and recessive aminoacyl-tRNA synthetase (ARS) disorders and the animal models that have been developed up to date.

In the first review, Kalotay et al. (a) focus on dominant ARS-disorders (ARSs), which have been linked to forms of peripheral neuropathy including Charcot-Marie-Tooth disease, distal hereditary motor neuropathy, and spinal muscular atrophy. Thus far, autosomal dominant mutations in *AARS1, GARS1, HARS1, MARS1, WARS1, SARS1*, and *YARS1* have been linked to peripheral neuropathies, predominantly Charcot-Marie-Tooth (CMT) disease and a variety of animal models is available, from drosophila to zebrafish to mouse. In this exhaustive review, the authors provide a list of the major models for dominant ARSs and highlight the importance of animal modeling, with a final reminder on how supplementing data obtained from animal studies with alternative approaches, such as patient-derived iPSC disease models, will increase their translational relevance.

The following and final review in this Research Topic, Kalotay et al. (b) focus on recessive ARSs, which instead cause a diverse range of multi-system disorders that affect different tissues. Neurodevelopment is impaired in most ARS-associated disorders. In addition to central nervous system defects, recessive ARSs commonly affect liver and lungs. Patients with recessive ARSs often present with encephalopathies, with variable involvement of peripheral systems. Many of these disorders cause severe disability, the comprehension of their pathogenesis is currently limited, no treatment is available, and therefore the importance of animal models is striking. In this comprehensive review, the authors discuss the challenges in generating animal models for these disorders, as well as key results and future directions.

This Research Topic provides an update on *in-vitro* and *in-vivo* models for NDDs, spanning from a new study on serotonergic matrix in brain development to the importance of iPSCs in Alzheimer's Disease and the wide aminoacyl-tRNA synthetase disorders field. Despite the efforts and progress from the scientific community in filling the gaps in the last years, many questions are still unresolved, many mechanisms need to be uncovered and pathways unearthed. Our aim is to help expand the knowledge on NDDs and we hope these articles inspire researchers to collaborate and further investigate the broad spectrum of NDDs to identify therapeutic targets and potential drugs to improve patients' lives.

## Author contributions

AD is the guest-topic editor and drafted and revised the editorial. MM, WA-S, and MH drafted and revised the editorial. All authors contributed to the article and approved the submitted version.

## References

[B1] Bozzi Y. Fagiolini M. (2020). Animal models of neurodevelopmental disorders. Neuroscience. 445, 1–2. 10.1016/j.neuroscience.2020.09.007 32967770

[B2] Cardoso A. R. Lopes-Marques M. Silva R. M. Serrano C. Amorim A. Prata M. J.. (2019). Essential genetic findings in neurodevelopmental disorders. Hum. Genom. 13, 31. 10.1186/s40246-019-0216-4 31288856 PMC6617629

[B3] Díaz-Caneja C. M. State M. W. Hagerman R. J. Jacquemont S. Marín O. Bagni C.. (2021). A white paper on a neurodevelopmental framework for drug discovery in autism and other neurodevelopmental disorders. Eur. Neuropsychopharmacol. (2021) 48:49–88. 10.1016/j.euroneuro.2021.02.020 33781629

[B4] Nussinov R. Yavuz B. R. Arici M. K. Demirel H. C. Zhang M. Liu Y.. (2023). Neurodevelopmental disorders, like cancer, are connected to impaired chromatin remodelers, PI3K/mTOR, and PAK1-regulated MAPK. Biophys. Rev. 15, 163–181. 10.1007/s12551-023-01054-9 37124926 PMC10133437

[B5] Parenti I. Rabaneda L. G. Schoen H. Novarino G. (2020). Neurodevelopmental disorders: from genetics to functional pathways. Trends Neurosci. (2020) 43, 608–621. 10.1016/j.tins.05004 32507511

[B6] Sabitha K. R. Shetty A. K. Upadhya D. (2020). Patient-derived iPSC modeling of rare neurodevelopmental disorders: Molecular pathophysiology and prospective therapies. Neurosci. Biobehav. Rev. (2021) 121, 201–219. 10.1016/j.neubiorev.12025 33370574 PMC7962756

[B7] Thapar A. Cooper M. Rutter M. (2017). Neurodevelopmental disorders. Lancet Psychiatry 4, 339–346. 10.1016/S2215-0366(16)30376-5 27979720

